# KOH-Assisted Molten Salt Route to High-Performance LiNi_0.5_Mn_1.5_O_4_ Cathode Materials

**DOI:** 10.3390/molecules30040797

**Published:** 2025-02-09

**Authors:** Feng Pang, Fushan Feng, Shuyu Zhang, Na Feng, Changkun Cai, Shengli An

**Affiliations:** 1 School of Rare Earth Industry, Inner Mongolia University of Science and Technology, Baotou 014010, China; pangfeng12138@163.com (F.P.); chinaxinl@126.com (S.Z.); fnn1018@163.com (N.F.); changkun_cai@126.com (C.C.); 2 Inner Mongolia Key Laboratory of Advanced Ceramic Materials and Devices, Inner Mongolia University of Science and Technology, Baotou 014010, China; 3 Key Laboratory of Green Extraction & Efficient Utilization of Light Rare-Earth Resources, Inner Mongolia University of Science and Technology, Ministry of Education, Baotou 014010, China

**Keywords:** lithium-ion batteries, LiNi_0.5_Mn_1.5_O_4_, cathode materials, KOH, high voltage

## Abstract

A simple and cost-effective route based on a KOH-assisted molten salt method is designed here to synthesize LiNi_0.5_Mn_1.5_O_4_ spinel. Pure-phase LiNi_0.5_Mn_1.5_O_4_ can be successfully prepared using chlorides as raw materials and adding KOH at 700 °C. The structure, morphology, and performance are discussed in detail. The measurements reveal that using KOH-assisted synthesis can optimize the crystal structure of the obtained LiNi_0.5_Mn_1.5_O_4_ samples, resulting in grain refinement while maintaining the predominantly octahedral structure that grows along the (111) crystal plane. This new synthesis pathway provides excellent performance in terms of cycle life. Electrochemical tests show that the KOH-assisted sample exhibits higher initial specific capacities (124.1 mAh·g^−1^ at 0.2 C and 111.4 mAh·g^−1^ at 3 C) and superior cycling performances (capacity retention of 85.0% after 200 cycles at 0.2 C and 95.70% after 100 cycles at 3 C). This provides a potential solution for the practical application of high-voltage LiNi_0.5_Mn_1.5_O_4_ lithium-ion batteries.

## 1. Introduction

Faced with the finite nature and the long-term environmental hazards of traditional hydrocarbon fuels, there is an urgent global demand for the development of clean and sustainable alternative energy technologies. In this context, renewable energy sources such as solar and wind power are particularly noteworthy. However, due to their intermittent nature, the efficient storage of electrical energy is crucial [1,2,3,4]. Lithium-ion batteries (LIBs), with their high energy density, low self-discharge rate, and long lifespan, have rapidly expanded in application, providing a strong impetus for the widespread adoption of renewable energy devices [5]. They have become the primary energy supply devices in fields such as portable electronic devices, electric vehicles (EVs), and aerospace applications [6]. It is projected that by 2045, the global installed capacity of lithium-ion batteries will reach 1500 GWh [7]. Therefore, further enhancement of lithium-ion battery performance is essential to meet the functional demands of the current market. Previous research indicates that the specific capacity, cycle life, safety, and cost of batteries are primarily influenced by cathode materials.

Currently, commercial lithium-ion battery cathode materials are mainly of two types: (i) layered structure LiCoO_2_ and its derivatives, such as LiNi_1−y−z_Mn_y_Co_z_O_2_ (NMC) and LiNi_1−y−z_Co_y_Al_z_O_2_ (NCA), and (ii) olivine-structured polyanion LiFePO_6_ [8,9,10,11]. It is noteworthy that the increasing price, low abundance, and toxicity of Co have hindered the widespread application of LiCoO_2_-based cathodes. Additionally, polyanion LiFePO_4_ materials suffer from low electrical conductivity and low discharge voltages [12]. Compared to these widely used cathode materials, spinel-type materials can provide three-dimensional ion channels for rapid Li^+^ diffusion, exhibiting superior rate capabilities during charge and discharge processes [13]. However, their commercialization has been relatively delayed. The manganese spinel cathode LiMn_2_O_4_ has been commercialized in first-generation plug-in hybrid electric vehicles (PHEVs) and pure electric vehicles (EVs), but its performance has been mixed [14]. Although this material has become a research hotspot, further improvements in its endurance and power performance are required in order to meet public demands and expand its application scope [14,15,16]. Nickel-substituted “high-voltage” spinel cathode LiNi_0.5_Mn_1.5_O_4_ is one of the most promising candidate materials. Compared to pure manganese spinel LiMn_2_O_4_, LiNi_0.5_Mn_1.5_O_4_ offers higher capacities (146.7 mAh·g^−1^) and operating voltage (4.7 V vs. Li^+^/Li), along with relatively benign constituent elements, making it one of the preferred materials for next-generation high-power batteries [17]. Despite these attractive features, the material still faces significant commercialization challenges. Due to the coupled redox potentials of Ni^2+^/Ni^3+^ and Ni^3+^/Ni^4+^, most of the battery’s electrochemical reactions occur around 4.7 V [18]. While this high potential can enhance power density, it also exacerbates undesirable side reactions between the active cathode material and the electrolyte solution. This results in a substantial thickening of the solid electrolyte interphase (SEI) layer, which hinders lithium-ion diffusion. Additionally, the interaction between Mn^4+^ and Ni^2+^ ions within the crystal structure adds another layer of complexity. Mn^4+^ and Ni^2+^ ions tend to order at the 16d octahedral sites of the spinel lattice: (Li)_8a_[Ni_0.5_Mn_1.5_]_16d_O_4_. Different synthesis routes or conditions can lead to variations in the Ni/Mn ratio and the degree of cation ordering, ranging from completely random distributions to highly ordered structures. The prevalence of cation ordering can appear anywhere in the spectrum. Other chemical modifications (such as doping) and physical modifications (such as surface morphology design) can also profoundly impact electrochemical performance, including cycle life and rate capability [19]. The molten salt synthesis method, which uses low-melting-point salts or mixtures of salts as the reaction medium, has been employed as an effective approach to precisely control the properties of LNMO materials. This method can enhance the crystallinity of the final product, and the particle size, morphology, degree of ordering/disordering, and Mn^3+^ content can be adjusted by varying synthesis conditions [20,21,22]. Wan et al. [23] employed a molten-salt-assisted method using molten lithium molybdate (Li_2_MoO_4_) as the medium for ion diffusion and crystal growth to prepare large-sized (median grain size D50 = 16.8 μm) single-crystal LNMO. The optimized LNMO sample exhibited excellent cycling performance, retaining approximately 85% of its initial capacity after 300 charge–discharge cycles. However, due to the larger particle size of the material, its rate performance was relatively poor, indicating the need for further research and improvement. Spence et al. [24] employed the molten salt synthesis method to design LNMO materials with specific crystal planes and a controllable Mn^3+^ content. Through post-treatment techniques, impurities were reduced to enhance electrochemical performance. The sample synthesized at 650 °C under a salt flux ratio of 80:20 in LiCl–KCl exhibited the best overall performance, with a maximum discharge capacity of 115.8 mAh·g^−1^ and a capacity retention of 96.8% after 100 cycles. Its rate performance exhibited a capacity of 84 mAh·g^−1^ at a 10 C rate.

In this study, pure-phase LiNi_0.5_Mn_1.5_O_4_ with a Fd-3m structure was successfully synthesized via the KOH-assisted in situ molten salt method at 700 °C. The resulting materials exhibited a well-dispersed octahedral morphology with micron-sized particles. The effect of the KOH additive on the electrochemical performance was investigated, and the particle morphology and crystal structure of different samples were compared, demonstrating the influence of crystal structure and microstructure on electrochemical performance. These findings provide new insights for future research on high-voltage lithium-ion batteries.

## 2. Results and Discussion

The spinel structure of LiNi_0.5_Mn_1.5_O_4_ can be described as a cubic close packing of oxygen atoms, with manganese (Mn) and nickel (Ni) ions occupying half of the octahedral sites and lithium (Li) ions occupying one-eighth of the tetrahedral sites. Specifically, in the crystal structure, Mn and Ni ions are ordered in the octahedral sites, corresponding to the P4_3_32 space group. This ordered structure results in a regular arrangement of Mn and Ni within the lattice. Conversely, when Mn and Ni ions are randomly distributed in the octahedral sites, the structure corresponds to the Fd-3m space group. This disorder results in a more random distribution of Mn and Ni within the spinel framework. In the ordered P4_3_32 phase, more detailed information peaks can be observed due to the ordering of manganese and nickel ions [25,26].

**Figure 1:**
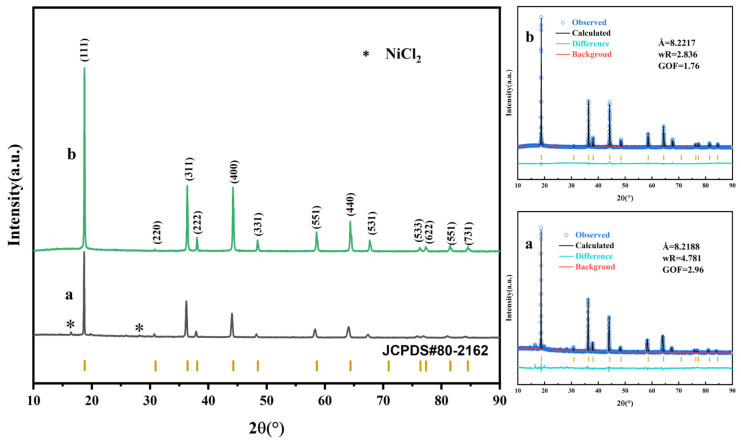
XRD patterns and Rietveld refinement results of the LiNi_0.5_Mn_1.5_O_4_ samples: (**a**) the pristine sample; (**b**) the KOH-assisted sample.

Figure 1 presents the XRD patterns of the LiNi_0.5_Mn_1.5_O_4_ samples. Both samples exhibit XRD patterns consistent with the Fd-3m space group, with sharp characteristic peaks appearing at (111), (311), (400), and (440), indicating a well-formed crystal structure that matches the standard diffraction pattern (JCPDS#80-2162) for LiNi_0.5_Mn_1.5_O_4_ materials with the disordered structure. In the original sample, impurity peaks corresponding to NiCl_2_ were observed, indicating that although chloride was used as a precursor to successfully synthesize the disordered LiNi_0.5_Mn_1.5_O_4_ at 700 °C, the process also resulted in the formation of impurity phases, which could degrade the material’s performance. In contrast, the KOH-assisted sample exhibits a pure phase with significantly enhanced diffraction peak intensities. The structural refinement of the two samples reveals that the lattice parameter of the pristine sample is 8.2188 Å, while that of the KOH-assisted sample is slightly increased to 8.2217 Å, which may be related to the formation of specific crystal planes.

**Figure 2:**
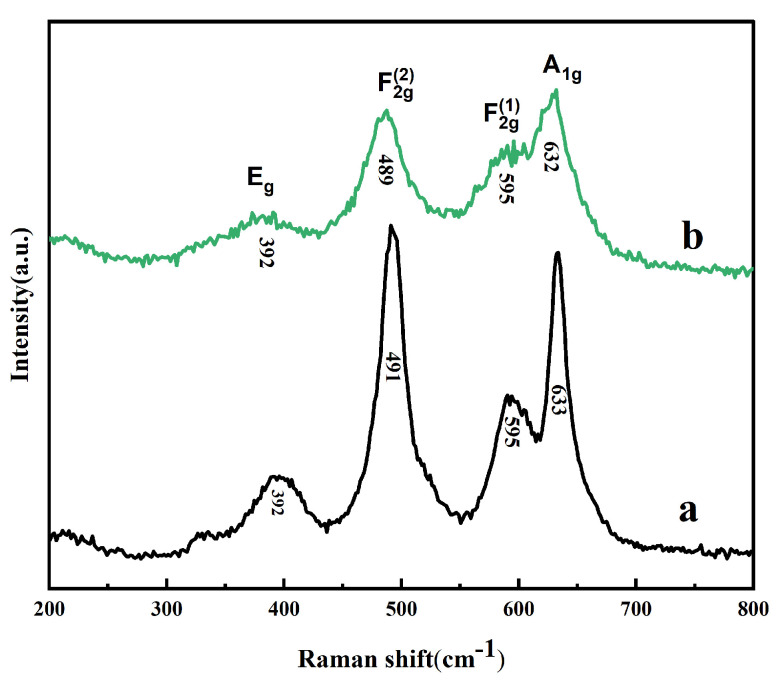
Raman spectra of the LiNi_0.5_Mn_1.5_O_4_ samples: (**a**) the pristine sample; (**b**) the KOH-assisted sample.

Raman spectroscopy was further adopted to identify the structure of our prepared LiNi_0.5_Mn_1.5_O_4_ samples, and the spectra are shown in Figure 2. The two samples have a similar Raman spectrum with the same number of peaks. According to previous reports, the peak near 636 cm^−1^ corresponds to Mn-O stretching, while the peaks near 399 and 496 cm^−1^ are attributed to Ni^2+^-O stretching [27,28]. The ordered phase is expected to exhibit more peaks in the spectrum compared to the disordered phase. The complete splitting of the band around 595 cm^−1^, along with the presence of sharp peaks at approximately 221 cm^−1^ and 241 cm^−1^, can be regarded as characteristic features of the ordered LiNi_0.5_Mn_1.5_O_4_ phase. Herein, these characteristics of ordered structure are not observed, suggesting that the two samples have a disordered structure. This observation is consistent with the sharper peaks observed in the XRD patterns. By comparing the Raman spectra and XRD patterns of different samples, it is evident that both samples exhibit the Fd-3m space group, primarily showing a disordered structure. This indicates that LiNi_0.5_Mn_1.5_O_4_ materials with an Fd-3m structure were successfully synthesized to achieve structural optimization.

**Figure 3:**
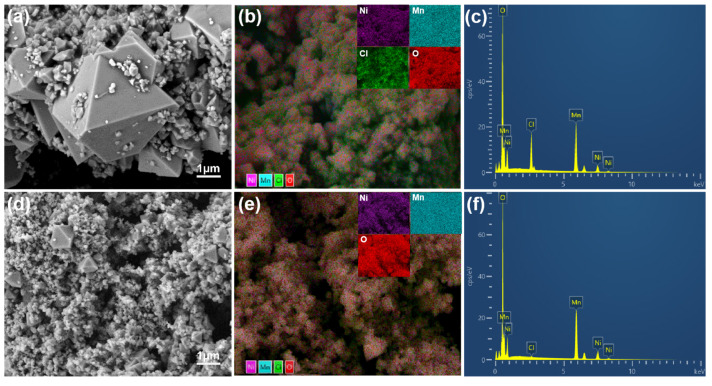
Scanning electron microscopy (SEM) images and energy-dispersive spectroscopy (EDS) analyses of the LiNi_0.5_Mn_1.5_O_4_ samples: (**a**–**c**) the pristine sample; (**d**–**f**) the KOH-assisted sample.

The cathode material surface morphology and particle size distribution are crucial factors influencing its electrochemical performance. Therefore, particle size and morphology analyses were conducted on various samples. As shown in Figure 3, the scanning electron microscope (SEM) image of the original sample is displayed, which exhibits a typical octahedral structure. However, the crystal structure of the original sample is incomplete, and secondary phases are present. The XRD pattern indicates that the secondary phase is NiCl_2_.

The KOH-assisted sample exhibits octahedral morphology, with the octahedral particles encapsulated by agglomerated material. Due to the addition of KOH, the previously distinct grains begin to coalesce, making them difficult to distinguish. This coalescence is attributed to the increasing aggregation of the material with the addition of KOH. During calcination, the removal of potassium ions causes the originally distinct crystal structures to merge into a tightly connected, unified structure, making its morphological features difficult to identify. Additionally, it was observed that the addition of KOH resulted in a decrease in grain size, while the secondary phase disappeared, resulting in the formation of a complete cubic structure with higher crystallinity and a more uniform grain size.

The EDS image clearly shows that in the original sample, the Cl element is distributed on the surface of part of the cathode material, indicating the presence of secondary phases or impurities in the original sample. Notably, by comparing the element distribution maps of the two samples (Figure 3c,f), it can be observed that the peak corresponding to Cl almost completely disappears after the addition of KOH, suggesting that the chloride impurity (NiCl_2_) in the sample has been largely removed.

**Figure 4:**
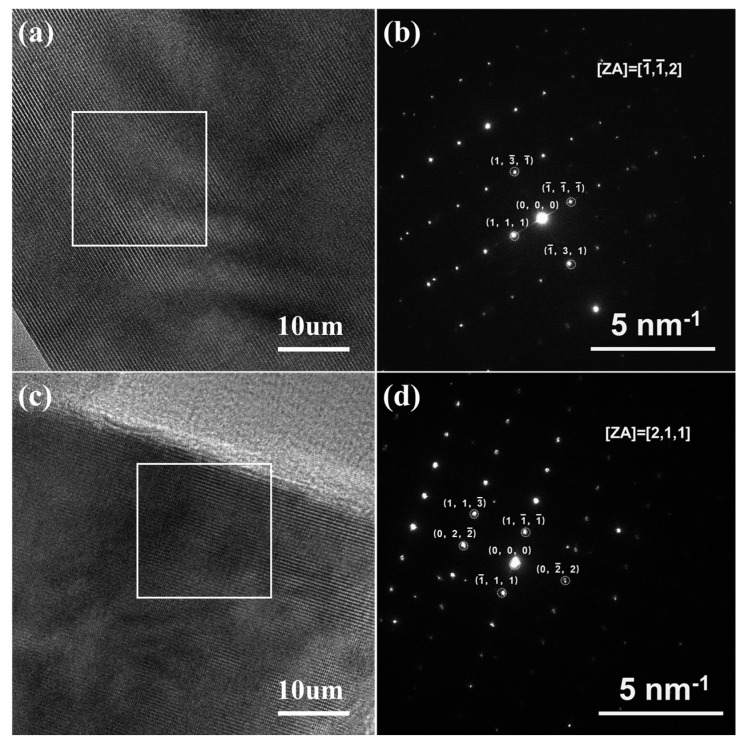
HR-TEM and selected-area electron diffraction (SAED) images of the LiNi_0.5_Mn_1.5_O_4_ samples: (**a**,**b**) the pristine sample; (**c**,**d**) the KOH-assisted sample (Panels **b** and **d** correspond to the white-framed areas in Panels **a** and **c**, respectively).

The microstructural changes in the LiNi_0.5_Mn_1.5_O_4_ material induced by the KOH treatment were investigated using high-resolution transmission electron microscopy (HRTEM) (Figure 4). The HRTEM images of both samples show smooth, clean surfaces and highly contrasted lattice fringes, indicating excellent crystallinity, well-preserved crystal structure, and minimal defects. A selected-area electron diffraction (SAED) analysis further confirmed that the diffraction spots of the original sample and the KOH-assisted sample correspond to the atomic arrangements of the LNMO materials with the Fd-3m space group along the [112] and [211] directions, respectively. The diffraction spots are clear and regular, and their clarity is related to the high degree of order in the crystal structure. This suggests that the KOH treatment has minimal impact on the lattice and does not alter its fundamental crystal structure or significantly disrupt the atomic arrangement, which is beneficial for achieving superior electrochemical performance.

In conclusion, the KOH treatment results in a single, intact octahedral crystallite characteristic of the spinel structure, with higher crystallinity and structural stability, significantly improving the electrochemical performance and cycling stability of the material.

As shown in Table 1, both the pristine and KOH-assisted samples exhibit relatively concentrated particle size distribution and high crystallinity, which is consistent with the XRD analysis results. The concentrated particle size distribution indicates minimal variation in particle size, contributing to a more uniform electrochemical reaction and enhancing the rate capability of the battery, thereby reducing the likelihood of particle agglomeration. It is worth noting that the width factor of the KOH-assisted sample is slightly higher, and this is possibly due to the minor agglomeration caused by the smaller grain size. However, the D10, D50, and D90 data indicate that the overall particle diameter of the KOH-assisted sample is smaller. In lithium-ion batteries, smaller particles typically have a larger specific surface area, which increases the active surface area of the electrode material, accelerates charge transfer, and improves ion diffusion. Furthermore, smaller and more uniform particles help reduce electrode polarization, lower resistance, and optimize electrode performance. This ultimately enhances the cycling stability and power density of the battery.

**Figure 5:**
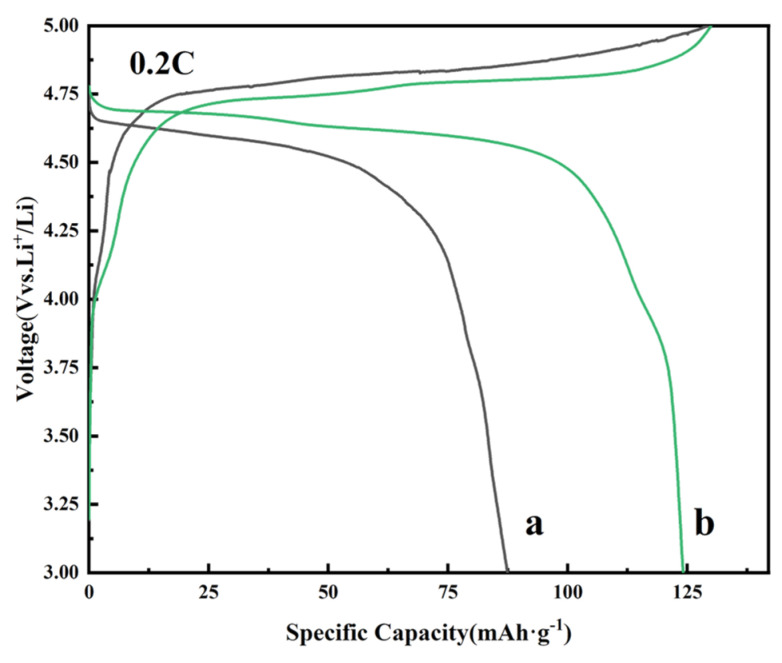
Charge–discharge curves of the LiNi_0.5_Mn_1.5_O_4_ samples at 0.2 C within the voltage range of 3.0–5.0 V: (**a**) the pristine sample; (**b**) the KOH-assisted sample.

To demonstrate that the addition of KOH can effectively enhance the electrochemical performance of the LiNi_0.5_Mn_1.5_O_4_ material, the pristine and KOH-assisted samples were selected as cathode materials to assemble coin cells. The initial charge–discharge curves of both LiNi_0.5_Mn_1.5_O_4_ samples are shown in Figure 5. The long, flat plateau at approximately 4.7 V represents the redox reactions involving Ni^2+^/Ni^3+^/Ni^4+^, while the plateau around 4.0 V can be attributed to the redox reactions of Mn^3+^/Mn^4+^ [29,30,31]. The 4.1 V plateau becomes more obvious (Figure 5b), which means an increase in the content of Mn^3+^ in the KOH-assisted sample. The discharge-specific capacities of the pristine sample and the KOH-assisted sample were 87.5 mAh·g^−1^ and 122.5 mAh·g^−1^, with Coulombic efficiencies of 77.66% and 87.88%, respectively. The results show that the KOH-assisted LiNi_0.5_Mn_1.5_O_4_ sample improves the initial discharge capacity and Coulombic efficiency, which should be associated with the increased content of Mn^3+^ that enhances the specific capacity of this obtained sample.

**Figure 6:**
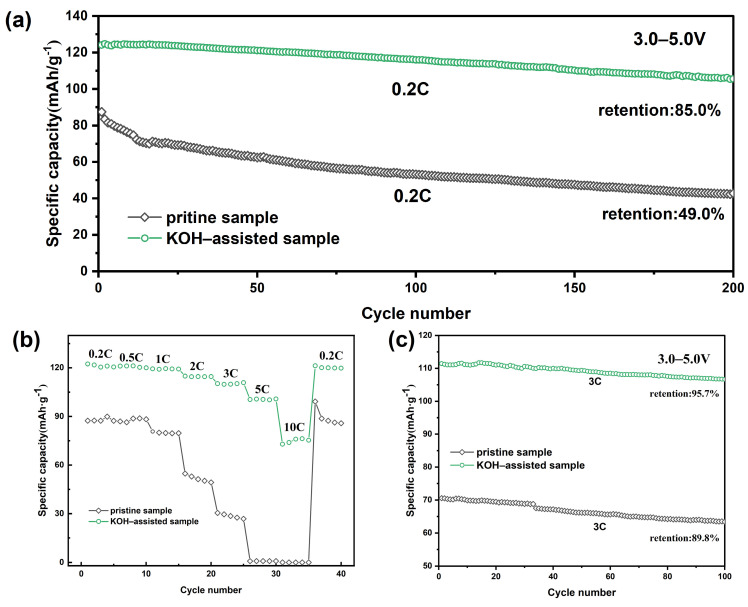
Electrochemical performance of LiNi_0.5_Mn_1.5_O_4_ samples: (**a**) cycling performance at 0.2 C rate; (**b**) rate performance; (**c**) cycling performance at 3 C rate.

Figure 6a shows the cycling performance of the LiNi_0.5_Mn_1.5_O_4_ material after 200 cycles at a 0.2 C rate. As shown, the KOH-assisted synthesized sample exhibits a higher cycling discharge capacity compared to the original sample under the same 0.2 C cycling rate. The initial discharge capacities of the original sample and the KOH-assisted synthesized sample were 87.4 mAh·g^−1^ and 124.1 mAh·g^−1^, respectively. After 200 cycles at 0.2 C, the discharge capacities of the original sample and the KOH-assisted sample were 42.8 mAh·g^−1^ and 105.5 mAh·g^−1^, with capacity retention rates of 49.0% and 85.0%, respectively. These results suggest that the addition of KOH during the synthesis of the LiNi_0.5_Mn_1.5_O_4_ precursor can enhance the cycling performance of the lithium nickel manganese oxide material. To further confirm the effect of KOH addition, the original sample and the KOH-assisted synthesized sample were tested at a 3 C rate (Figure 6c). The initial discharge capacities of the original sample and the KOH-assisted sample were 70.6 mAh·g^−1^ and 111.4 mAh·g^−1^, respectively. After 100 cycles, the capacity retention rates of the original sample and the KOH-assisted sample were 89.8% and 95.7%, respectively.

Figure 6b shows the rate performance of the obtained samples. The discharge capacity of both the pristine and the KOH-assisted samples decreases with an increase in current density. The discharge-specific capacity of the KOH-assisted sample is significantly higher than that of the pristine sample. At the 3 C rate, the discharge-specific capacity of the pristine sample is 28.6 mAh·g^−1^, while that of the KOH-assisted sample is 110.2 mAh·g^−1^. However, at a high rate of 10 C, the discharge-specific capacity of the pristine sample dropped to only 0.041 mAh·g^−1^ (nearly zero), whereas the KOH-assisted sample still retained a high discharge-specific capacity of 72.8 mAh·g^−1^. This indicates that the KOH-assisted sample can provide excellent electrochemical performance.

**Figure 7:**
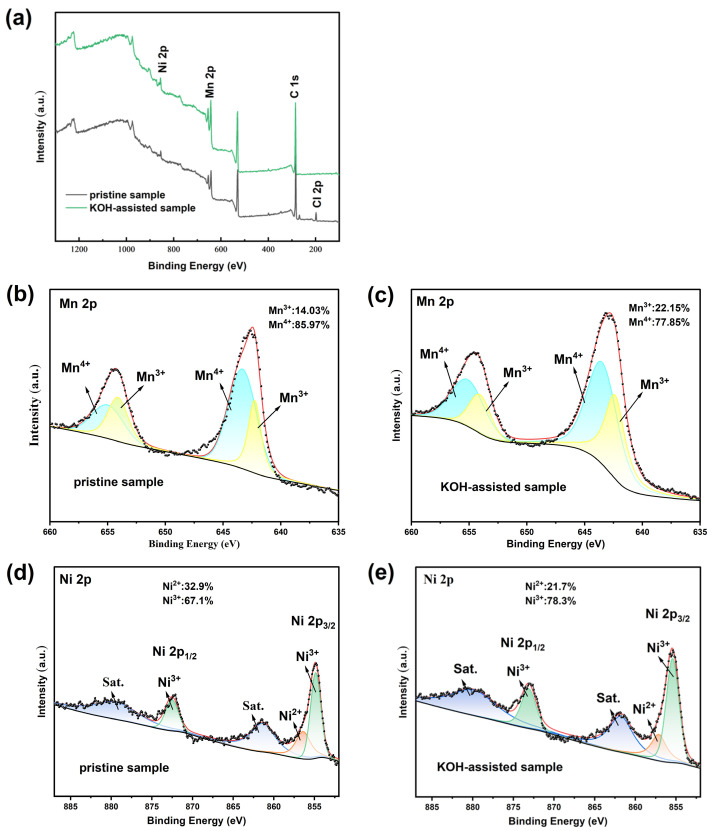
XPS spectra of LiNi_0.5_Mn_1.5_O_4_ samples: (**a**) wide scan range; (**b**,**c**) Mn 2_P_; (**d**,**e**) Ni 2_P_.

The valence state changes in the pristine and KOH-assisted samples were analyzed using X-ray photoelectron spectroscopy (XPS) (Figure 7). Figure 7 compares the XPS spectra of the pristine sample and the KOH-assisted sample. The full-spectrum analysis reveals that the spectra of both samples are essentially identical, indicating that the addition of KOH did not significantly alter the overall structure of the material. However, the pristine sample exhibits a Cl 2p peak, which is associated with the presence of NiCl_2_ as an impurity phase. In contrast, no such peak is observed in the KOH-assisted sample, further confirming that the KOH-assisted synthesis effectively removed the impurity phase. The XPS spectra of Ni and Mn elements in both samples show mixed valence states of Ni^2+^/Ni^3+^ and Mn^3+^/Mn^4+^. Specifically, the characteristic peaks for Mn^4+^ appear at 665.2 eV and 643.4 eV, while those for Mn^3+^ are observed at 654.1 eV and 642.2 eV. The Ni^2+^ peak is located at 872.6 eV, and the Ni^3+^ peak is observed at 854.9 eV. The relative concentrations of Mn^3+^, Mn^4+^, Ni^2+^, and Ni^3+^ in the two samples were determined by calculating the peak areas. The Mn^3+^ content in the original and KOH-assisted samples was 14.03% and 22.15%, respectively, while the Ni^3+^ content was 67.1% and 78.3%, respectively. These changes are consistent with the maintenance of the electrochemical neutrality of the samples. This study primarily focuses on the analysis of manganese valence state variations. However, excessive Mn^3+^ contents in LiNi_0.5_Mn_1.5_O_4_ materials can result in capacity fading and the degradation of electrode materials during cycling. In contrast, the KOH-assisted sample exhibited better electrochemical performance. This result contradicts the understanding that trivalent manganese negatively impacts the battery’s cycle life, as Mn^3+^ results in Jahn–Teller distortion and subsequent structural imbalance [32,33]. This phenomenon is attributed to the reversible utilization of the Mn^3+^/Mn^4+^ redox couple in a high-voltage spinel, and the appropriate amount of Mn^3+^ enhances the ion conductivity and charge transfer rate of the material, thereby improving its electrochemical performance [34,35,36,37]. Moreover, the first charge–discharge curves of the original sample and the KOH-assisted sample (Figure 5) indirectly support this, where the electrochemical plateau corresponding to the manganese element is around 4.1 V. The increase in Mn^3+^ content in the KOH-assisted sample results in an extension of the electrochemical plateau, thereby enhancing the specific capacity of the material.

**Figure 8:**
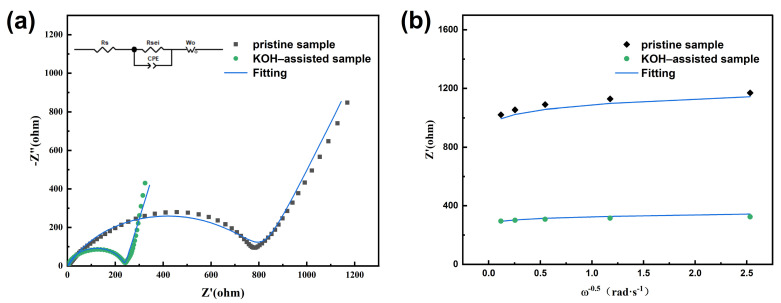
Impedance data of the LiNi_0.5_Mn_1.5_O_4_ samples: (**a**) impedance spectra; (**b**) fitting results of Z’ vs. ω^−0.5^ (slope: σ).

Figure 8 shows the electrochemical impedance spectra (EIS) of the LiNi_0.5_Mn_1.5_O_4_ samples. An EIS analysis was performed on the pristine and KOH-assisted samples to further compare the kinetic behavior of the crystal samples during the lithium extraction process.

As expected, the EIS plot exhibits a semicircle and a line representing the high-frequency and low-frequency ranges, respectively, along with the corresponding equivalent circuit diagram. The diameter of the semicircle is approximately equal to the charge transfer resistance (Rct), while the low-frequency region of the line is attributed to the diffusion of lithium ions within the electrode material [38]. Rsei corresponds to the diffusion resistance of Li^+^ ions on the surface of the active material, Rct is the charge transfer resistance at the electrode–electrolyte interface, and CPE represents the double-layer capacitance. The Z′ vs. ω^−0.5^ plot was obtained via impedance in the low-frequency region and according to Equations (1) and (2), as shown in Figure 8b. Since the specific value of Rsei cannot be directly obtained, the size of Rsei is indirectly represented by calculating the slope (δ) of Figure 8b [39,40]. The specific values of Rct and δ for each sample were obtained by fitting the impedance curves, as listed in Table 2. Based on the variations in the specific values of Rct and δ in Table 2 and the data presented in Figure 8, it is evident that the electrode of the KOH-assisted sample exhibits significantly lower Rct and Rsei values.

Simultaneously, by fitting the Z′ vs. ω^−0.5^ plot (Figure 8b), the Warburg impedance coefficient corresponding to the low-frequency region of the impedance curve can be obtained. Using Equations (1)–(3), the lithium-ion diffusion coefficient in the electrode material can be calculated [41,42,43,44]:

(1) ω=2πf

(2) $$Z^{\prime }=R_{s}+\delta \omega^{-0.5}$$

(3) $$D_{Li+}=\frac{R^{2}T^{2}}{2A^{2}n^{4}F^{4}C_{Li}^{2}\delta^{2}}$$

R denotes the ideal gas constant (R = 8.314 J/(mol·K)), Rs refers to the ohmic resistance, T indicates the thermodynamic temperature, A signifies the surface area of the cathode electrode (A = 1.5386 cm^2^), n is the number of electron transfers, F represents the Faraday constant, C_Li+_ is the molar concentration of Li^+^ ions in the material (C_Li+_ = 0.02378 mol·cm^−3^) [39,45]; and σ is the Warburg coefficient, and it reflects the linear relationship between the inverse square root of the angular frequency and the Warburg impedance (Figure 8b). Moreover, ω represents the angular frequency, which can be calculated using Equation (1).

The lithium-ion diffusion coefficient (D_Li+_), as shown in Table 2, indicates that the lithium-ion diffusion rate of the KOH-assisted samples is significantly enhanced. Smaller particles have a larger specific surface area and provide shorter ion migration pathways, which facilitates faster lithium-ion diffusion within the electrode material. Additionally, the presence of an appropriate amount of Mn^3+^ further enhances the ionic conductivity and charge transfer rate of the material. Mn^3+^ helps regulate the charge transport pathways, promoting more efficient lithium-ion migration within the electrode and further improving the electrochemical performance of the material.

## 3. Materials and Methods

### 3.1. Synthesis Procedure

**Figure 9:**
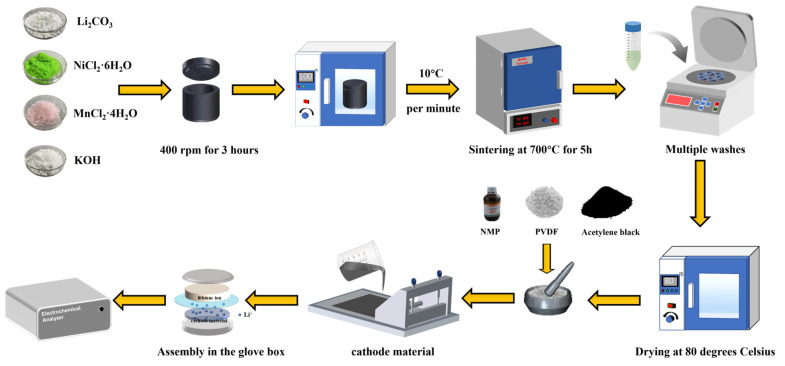
Schematic diagram of the preparation process for LiN_i0.5_Mn_1.5_O_4_ samples.

In this study, all the chemical reagents used were of analytical reagent (A.R.) grade. LiNi_0.5_Mn_1.5_O_4_ powder was synthesized via an in situ molten salt method. Specifically, Li_2_CO_3_, NiCl_2_·6H_2_O, MnCl_2_·4H_2_O, and KOH (with a cation molar ratio of Li:Ni:Mn = 1:0.5:1.5, and KOH calculated based on the molar mass of MnCl_2_, corresponding to either 1.5 mol or no addition of KOH) were placed in an agate jar and ball-milled (400 r/min for 3 h) using a planetary ball mill. After milling, the mixture was dried in a vacuum oven at 110 °C for 12 h. The dried material was then pulverized and further dry-milled for 20 min to form the precursor. Subsequently, an appropriate amount of the precursor was transferred to a crucible and calcined in a muffle furnace at 700 °C in the air for 5 h. After calcination, the sample was allowed to naturally cool below 80 °C. The resulting sample was then added to deionized water and placed in a centrifuge at a speed of 6000 rpm for 30 min to remove K^+^ ions. Finally, the sample was dried in an oven at 110 °C for 12 h to obtain the LiNi_0.5_Mn_0.5_O_4_ product. The preparation method for the pristine and KOH-assisted samples is identical, except that no KOH reagent was added in the former. The schematic diagram of the entire preparation process for LiN_i0.5_Mn_1.5_O_4_ is shown in Figure 9.

### 3.2. Characterizations of LiNi_0.5_Mn_1.5_O_4_ Powder

The obtained LiNi_0.5_Mn_1.5_O_4_ samples were characterized via X-ray diffraction (XRD, Bruker-D8 Advance, Karlsruhe, Germany) with a scanning range of 10–90° and a scanning speed of 10°/min. The nature of chemical bonds and the crystalline characteristics of the LiNi_0.5_Mn_1.5_O_4_ particles were measured via a Raman spectrometer (iHR550, HORIBA, Kyoto, Japan) with a 532 nm laser over the range of 200–800 cm^−1^. The microstructures were characterized via a field emission scanning electron microscope (FESEM, Carl Zeiss-Sigma300, Jena, Germany) and transmission electron microscopy (TEM, FEI G2-300, Hillsboro, OR, USA). The average oxidation state of Mn was determined through XPS measurement (K-Alpha, Thermo Fisher Scientific, Waltham, MA, USA) using monochromatic Al Kα (1486.6 eV) radiation under a vacuum of 1.0 × 10^−8^ mbar. The particle size distribution of the samples was measured using a Coulter LS230 (Brea, CA, USA) laser diffraction particle size analyzer. The samples were sonicated for 10 min in water, with a light absorption rate of 10^4^. Sodium hexametaphosphate was used as the dispersing agent. The refractive index of the sample was set to 2, and the refractive index of the medium was 1.333.

### 3.3. Electrochemical Tests

The working electrode was prepared using LiNi_0.5_Mn_1.5_O_4_ material, acetylene black, and polyvinylidene fluoride (PVDF) binder at a mass ratio of 8:1:1. These components were uniformly mixed with an appropriate amount of N-Methyl-pyrrolidone (NMP) to form a slurry. The slurry was uniformly coated onto aluminum foil using a coating machine. The coated aluminum foil was dried in a vacuum oven at 100 °C for 12 h to form cathode sheets with a diameter of 14 mm and an active material loading of 3.0–4.5 mg. Lithium metal and a Celgard 2400 polypropylene microporous membrane were used as the counter electrode and separator, respectively. The electrolyte used was 1 mol/L LiPF_6_ (in a volume ratio of EC:DMC:EMC = 1:1:1). The Li/LiNi_0.5_Mn_1.5_O_4_ half-cells (CR2025 coin cells) were assembled in an argon-filled glove box. The assembled cells were left to stand for 12 h in order to ensure the complete saturation of the electrolyte before conducting electrochemical tests. The current density of the samples was calculated based on the mass of the active material and the cell testing system (CT-4008Tn). The cycling voltage range of the cells was 3.0–5.0 V (vs. Li^+^/Li), and the current rate was maintained at 0.2–10 C (1 C = 147 mAh·g^−1^). Electrochemical tests were conducted at 25 °C to evaluate the discharge capacity, cycling performance, and rate capability.

## 4. Conclusions

High-crystallinity, pure-phase spinel-type LiNi_0.5_Mn_1.5_O_4_ material with octahedral morphology could be easily synthesized using a KOH-assisted in situ molten salt method at 700 °C. The structure, morphology, kinetics, and electrochemical performance of spinel LiNi_0.5_Mn_1.5_O_4_ cathode materials were compared in detail. The results indicate that chlorination of the raw materials primarily produced LiNi_0.5_Mn_1.5_O_4_ with a disordered structure at 700 °C, but impurity phases negatively affected its electrochemical performance. The introduction of KOH as an additive eliminated these impurities, optimized the crystal structure of the LiNi_0.5_Mn_1.5_O_4_ samples, and reduced the grain size. The KOH-assisted sample assistance exhibited an initial discharge capacity of 124.1 mAh·g^−1^ at 0.2 C, with a capacity retention of 85.0% after 200 cycles. At the 3 C rate, the initial discharge capacity was 110.2 mAh·g^−1^, with a capacity retention of 95.7% after 100 cycles. Furthermore, at a high rate of 10 C, the discharge capacity remained at 72.8 mAh·g^−1^. Simultaneously, using the KOH-assisted in situ molten salt method enhanced the structural stability of the LiNi_0.5_Mn_1.5_O_4_ and simplified the entire synthesis process. This novel synthesis method lays a solid foundation for further cost effectiveness and introduces a promising pathway for the development and commercialization of high-voltage lithium-ion batteries.

## Figures and Tables

**Table 1:** Particle size analysis of the LiNi_0.5_Mn_1.5_O_4_ samples: (a) the pristine sample; (b) the KOH-assisted sample.

Samples	D10 (μm)	D50 (μm)	D90 (μm)	Span (Span = (D90−D10)/D50)
a	3.47	26.0	65.7	2.39
b	3.18	13.9	38.9	2.57

**Table 2:** The electrochemical impendence fitting results and calculated values of D_Li+_ of the LiNi_0.5_Mn_1.5_O_4_ samples: (a) the pristine sample, (b) the KOH-assisted sample.

Samples	Rs (Ω)	Rct (Ω)	δ (Ω·s^0.5^)	D_Li_ + (cm^2^·s^−1^)
a	4.178	788.364	97.629	2.770 × 10^−13^
b	2.376	235.032	31.331	8.444 × 10^−13^

## Data Availability

The data presented in this study are available on request from the corresponding author.
